# Psychological distress and tumor cell β_2_-adrenergic receptor levels in patients with surgically resected non-small cell lung cancer

**DOI:** 10.2340/ao.v65.45317

**Published:** 2026-05-04

**Authors:** Hronn Hardardottir, Thor Aspelund, Bryndis Valdimarsdottir, Jurate Asmundsson, Vigdis Petursdottir, Katja Fall, Erika Broström, Heiddis Valdimarsdottir, Fang Fang, Magnus K. Magnusson, Thorarinn Gudjonsson, Tomas Gudbjartsson, Erica K. Sloan, Susan K. Lutgendorf, Christer Janson, Unnur A. Valdimarsdottir

**Affiliations:** a Centre of Public Health Sciences, Faculty of Medicine, University of Iceland, Reykjavik, Iceland; b Department of Respiratory Medicine, Landspitali University Hospital, Reykjavik, Iceland; c Stem Cell Research Unit, BioMedical Center, School of Health Sciences, University of Iceland, Reykjavík, Iceland; d Department of Pathology, Landspitali University Hospital, Reykjavik, Iceland; e Clinical Epidemiology and Biostatistics, School of Medical Sciences, Örebro University, Örebro, Sweden; f Institute of Environmental Medicine, Karolinska Instituted, Stockholm, Sweden; g Department of Immunology, Genetics and Pathology, Clinical and experimental pathology, Uppsala University, Uppsala, Sweden; h Department of Medical Sciences, Respiratory-, Allergy and Sleep Research, Uppsala University, Uppsala, Sweden; i Department of Psychology, Reykjavik University, Reykjavik, Iceland; j Department of Population Health Science and Policy, Icahn School of Medicine at Mount Sinai, New York, NY, USA; k Department of Medicine, Faculty of Medicine, University of Iceland, Reykjavík, Iceland; l deCODE Genetics/Amgen Inc., Reykjavík, Iceland; m Department Laboratory Hematology, Landspitali University Hospital, Reykjavik, Iceland; n Department of Cardiothoracic Surgery, Landspitali University Hospital, Reykjavik, Iceland; o Monash Institute of Pharmaceutical Sciences, Monash University, Parkville, Australia; p Department of Psychological and Brain Sciences, University of Iowa, Iowa City, IA, USA; q Department of Epidemiology, Harvard T.H. Chan School of Public Health, Boston, MA, USA

**Keywords:** psychological distress, lung cancer, β_2_-adrenergic receptors, sympathetic nervous system activation

## Introduction

Patients diagnosed with cancer often experience significant levels of psychological distress [1, 2], which has been associated with cancer progression [3–6] and increased cancer-specific mortality across different tumor types, including lung cancer [2, 7]. One potential mechanism by which psychological distress may affect cancer progression is through dysregulation of the hypothalamic–pituitary–adrenal axis and the sympathetic nervous system, which controls the release of catecholamines [8, 9]. The influence of catecholamines on tumor growth occurs in part through β-adrenergic receptors (β-ARs), which regulate multiple cellular processes, including tumor cell proliferation and invasion, and vascular integrity within tumors (angiogenesis) [3, 6, 10, 11]. The beta-2-adrenergic receptor (β_2_AR) subtype predominates in the lung (70%) [12], and signaling through β_2_AR has been associated with cancer progression through effects on tumor cell invasion [5, 13–17] and angiogenesis [6, 18].

While clinical studies are scarce, a recent publication indicates an association between symptoms of psychological distress and increased β_2_AR levels in patients with oral squamous cell carcinoma [19], and two clinical studies have associated β_2_AR expression with factors predicting poor outcomes in lung cancer [14, 15].

Therefore, in a patient population operated for newly diagnosed non-small cell lung cancer, we aimed to explore the association between psychological distress at diagnosis and tumor levels of β_2_AR.

### Patients/material and methods

The LUng CAncer, Stress and Survival (LUCASS) study is a prospective cohort study in Iceland and Sweden with the overarching aim to investigate the psycho-biological stress response in patients undergoing a diagnostic work-up for suspected lung cancer [20, 21]. Details on LUCASS can be found in our earlier publications [20, 21].

The analytic cohort comprised 52 LUCASS participants with non-small cell lung cancer (NSCLC) who underwent curative-intent surgical resection. Data on age, education, marital status, employment, financial and smoking status, and perceived psychological distress were collected and integrated with clinical evaluations conducted at two time points: before and after the final diagnosis of lung cancer. Perceived psychological distress, anxiety, and depression were assessed using the 14-item Hospital Anxiety and Depression Scale (HADS) [22], a widely used screening tool in oncology. According to established guidelines, HADS total (HADS-T) scores ≥ 13 indicated potentially clinically significant psychological distress, HADS anxiety (HADS-A) scores ≥ 7 indicated clinically significant anxiety, and HADS depression (HADS-D) scores ≥ 5 indicated clinically significant depression [23].

Although HADS was assessed at two time points, the present analyses used the first assessment, administered before a definitive lung cancer diagnosis was established. Where pre-diagnostic measures from questionnaires were not available (for six participants, 11.5%), primary analyses used post-diagnostic measures (but pre-surgical).

The time interval from HADS assessments before diagnosis to surgery was at a median of 42 days (mean 50.7, SD 30.5, range 8–163 days).

The surgery was performed at a median of 28.5 days after the confirmed diagnosis of lung cancer (mean 31.8, SD 29.8, range 0–111 days). Pathologic stages were determined according to the 8th Tumor, Node, Metastasis (TNM) classification system [24].

The lung cancer diagnosis was based on pathological confirmation according to international guidelines [25]. The date of diagnosis was defined as the date of the first reported pathology diagnosis. Paraffin-embedded tumor specimens were classified according to the World Health Organization (WHO) classification of lung cancer from 2021 [26], as adenocarcinoma in 33 (63.5%) and non-adenocarcinoma in 19 (36.5%).

**Figure 1:**
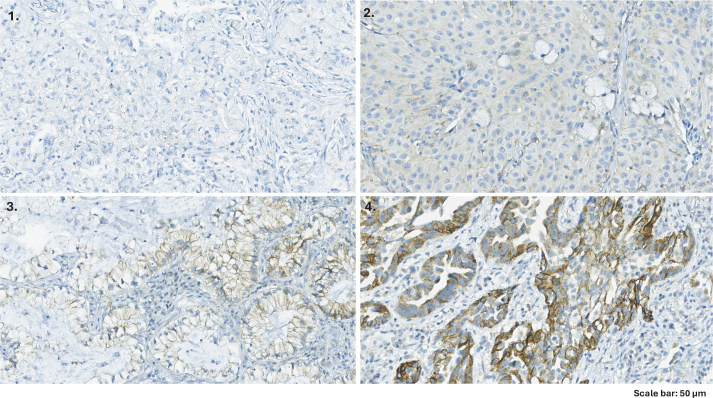
Representative β_2_AR immunostaining in NSCLC samples. Samples were defined as low β_2_AR levels if they had (1) < 10% or (2) 11–25% of tumor cells positive for β_2_AR. Samples were defined as high β_2_AR levels if they had (3) 26–50% or (4) > 50% of tumor cells positive for β_2_AR. NSCLC: Non-Small Cell Lung Cancer; β_2_AR: beta-2-adrenergic receptor.

β_2_AR expression was determined by immunohistochemical staining using rabbit antihuman β_2_AR monoclonal antibody (ab182136, Abcam, Inc., Cambridge, UK; 1:1000 dilution). The expression of β_2_AR was considered positive if distinct membrane and cytoplasmic staining were present [14]. β_2_AR expression levels were assessed based on the extent of the staining as follows: 1: ≤ 10% of the tumor cells stained; 2: 11–25% of the tumor cells stained; 3: 26–50% of the tumor cells stained; and 4: ≥ 51% of the tumor cells stained (Figure 1). β_2_AR expression levels ≥ 3 were defined as high β_2_AR levels [27].

Immunostaining was assessed using light microscopy in a blinded fashion by two experienced pathologists (JA and VP). In case of discrepancies, both pathologists evaluated the slides simultaneously until a consensus was reached.

### Statistical analysis

Summary statistics were used to describe the demographic and clinical characteristics of the study population. Predictive mean matching [28] was used to impute missing data in the HADS questionnaire (2.2%). Multiple logistic regression models were used to estimate the association between levels of β_2_AR and distress. All regression models were run without covariates and then including age and sex.

We performed all statistical analyses in R, version 4.3.1 [29] and the statistical significance was set at 0.05. This study and its procedures were approved by the Icelandic Bioethics Committee (VSNb201460025/03.07).

## Results

The cohort included 52 patients (50% women, mean age 69.5 years (SD 8.1)) who underwent surgical treatment for NSCLC, 33 (63.5%) with adenocarcinoma, and 24 (46.2%) with p-TNM stage IA. Distressed patients at diagnosis (HADS-T scores ≥ 13) were younger compared to those reporting lower distress (HADS-T scores < 13) (Supplementary Table 1). The levels of β_2_AR on cancer cells were high in 10 participants (19.2%): 59.3% of patients with high psychological distress (HADS-T ≥ 13) and 10.4% in patients who were not distressed (HADS-T < 13) (Table 1). When adjusting for age and sex, a strong positive association was observed between perceived psychological distress (HADS-T score ≥ 13) and β_2_AR levels on the cancer cells (odds ratio [OR] 12.6, 95% confidence interval [CI] 2.1–108, *p* = 0.010). Depressive symptoms (HADS-D score of ≥ 5) were positively associated with β_2_AR levels (OR 6.5, 95% CI 1.4–47.3, *p* = 0.028) while the association between anxiety (HADS-A score ≥ 7) and β_2_AR levels was non-significant (OR 4.1, 95% CI 0.7–29.3, *p* = 0.132) (Table 1).

**Table 1:** Association^a^ between psychological distress and tumor cell β_2_AR levels.

Psychological distress	% patients with high tumor cell β_2_AR levels^b^	OR^a^	95%CI^c^	*p*-value^d^
HADS^e^-T score				
< 13	10.4	Ref		
≥ 13	59.3	12.6	2.1–108	0.010
HADS^e^-D score								
< 5	7.1	Ref		
≥ 5	33.4	6.5	1.4–47.3	0.028
HADS^e^-A score								
< 7	13.7	Ref		
≥ 7	39.5	4.1	0.7–29.3	0.132

a Multiple regression analysis, adjusted for age and sex;

b Adjusted for age and sex;

c Confidence interval;

d *P*-values based on Chi-squared test when expected cell counts are less than 5;

e Hospital anxiety and depression scale. β2AR: beta-2-adrenergic receptor; OR: Odds Ratio; CI: Confidence Interval; HADS: Hospital Anxiety and Depression Scale.

Similar results were observed after excluding: (1) the six patients who answered the HADS after the cancer diagnosis (Supplementary Table 3), (2) the four patients who underwent neo-adjuvant treatment either concurrent chemo- or radio-therapy or surgery of a single metastasis in the brain or adrenal gland (Supplementary Table 4), or (3) the 11 patients with pTNM stages III–IV (Supplementary Table 5).

## Discussion and conclusion

The findings of this study suggest an association between a patient’s psychological distress before lung cancer diagnosis and β_2_AR levels on NSCLC cells at surgery. This was reflected in a strong association between levels of depression and tumor cell β_2_AR levels.

Inclusion of patients who were eligible for surgery with curative intent, limited the study to patients with low TNM staging. Consequently, the rates of psychological distress before surgery were lower in this patient cohort (19.2%) than previously reported in the total LUCASS population that included patients on all TNM stages and of all lung cancer histology types (30.1%) [20]. Despite the relatively low rate of psychological distress, our findings suggest that pre-diagnosis distress levels are associated with tumor cell β_2_AR levels in NSCLC.

Although the mechanism of how psychological distress may influence the regulation of β_2_AR on cancer cells remains unclear, studies have demonstrated that psychological distress activates the sympathetic nervous system [6, 30–32], which drives cancer progression [5, 6, 11, 13, 17, 18, 33–36] and is associated with poorer survival in lung cancer [37]. Our findings extend recent observations that mental health symptoms independently predicted tumor cell β_2_AR levels in patients with oral squamous cell carcinoma [19]. Furthermore, two previous clinical lung cancer studies [14, 15] reported an association between β_2_AR levels and tumor cell proliferation.

While larger studies are needed to verify our findings, to our knowledge, this study provides the first evidence that pre-diagnostic perceived psychological distress is associated with lung cancer cell β_2_AR levels. If confirmed, β_2_AR may be a novel target for treatment; indeed, some studies [37], but not all [38], suggest that blocking β-adrenoceptor activity may be associated with improved clinical outcomes in lung cancer [37].

The strength of this study includes the well-defined protocol for patients referred for lung cancer work-up, as well as the prospective assessment of patient distress levels before the diagnosis of lung cancer. The main limitation is the small study sample size (*n* = 52), with 19.2% of the patients having high levels of β_2_AR on the cancer cells, which limits the statistical power for several analyses. Furthermore, the distress assessment (HADS questionnaire) was made before lung diagnosis and at a median of 42 days before the surgery used to collect tumor for β_2_AR analysis, which may have influenced the association of these two measures. Finally, the study population is limited to patients diagnosed with NSCLC going through a diagnostic work-up at a tertiary care university hospital in Reykjavik, Iceland, and therefore, may not be readily generalized to other hospitals or countries.

In conclusion, this study showed a strong association between pre-diagnostic distress levels and cancer cell β_2_AR levels in patients undergoing curative surgical resection for lung cancer. Subject to replication in larger independent samples, these findings highlight the importance of surveillance for mental health symptoms in this vulnerable patient population, given the established role of β_2_AR signaling in cancer progression.

## Supplementary Material



## Data Availability

The authors have full access to the primary data and can make the data available to the journal or interested researchers upon approval of the National Bioethics Committee of Iceland.
