# Supplementary material for: Psychological distress and tumor cell β2-adrenergic receptor levels in patients with surgically resected non-small cell lung cancer
Source: Acta Oncol. 2026 May 4;65:45317. doi: 10.2340/ao.v65.45317 (PMC13151781; doi:10.2340/ao.v65.45317)
Supplement: Supplementary file 1 [file AO-65-45317-s1.pdf]

Supplementary material has been published as submitted. It has not been copyedited, or typeset by Acta Oncologica

Supplementary Table 1. Baseline characteristics of study participants by the level of psychological distress (HADS[1]-total score) before diagnosis of non-small cell lung cancer (NSCLC).

		**All** N (%)	**Low distress** N (%)	**High distress** N (%)	**p-value**[2]
**Overall** (N (%))		52	42(80.8)	10(19.2)	
Age	(years, mean (SD))	69.5(8.1)	71.0(7.6)	63.3(7.6)	0.006
Age at diagnosis	65 years and under	16(30.8)	10(62.5)	6(35.5)	0.052
	66-70 years	9(17.3)	7(77.8)	2(22.2)	
	71 years and above	27(51.9)	25(92.6)	2(7.4)	
Sex	male	26(50.0)	23(88.5)	3(11.5)	0.291
	female	26(50.0)	19(73.1)	7(26.9)	
Marital status	single/divorced/widowed	17(32.7)	14(82.4)	3(17.6)	1.000
	married/partnered	35(67.3)	28(80.0)	7(20.0)	
Educational level	primary	23(44.2)	17(73.9)	6(26.1)	0.533
	secondary	21(40.4)	18(85.7)	3(14.3)	
	university	8(15.4)	7(87.5)	1(12.5)	
Occupation	unemployed/retired	34(65.4)	27(79.4)	7(20.6)	1.000
	working	18(34.6)	15(83.3)	3(16.7)	
Financial status	enough/bad	24(65.4)	16(66.7)	8(33.3)	0.477
	very good/good	28(53.8)	26(92.9)	2(7.1)	
Smoking status	never/previous	25(48.1)	23(92.0)	2(8.0)	0.128
	current	27(51.9)	19(70.4)	8(29.6)	
Use of beta-blockers	yes	6(11.5)	4(66.7)	2(33.3)	0.703
Use of beta-agonists	yes	19(36.5)	15(78.9)	4(21.1)	1.000
	Other	3(5.8)	3(100)	0	
**Tumor characteristics**					
Histology	adenocarcinoma	33(63.5)	26(78.8)	7(21.2)	0.910
	non-adenocarcinoma	19(36.5)	16(84.2)	3(15.8)	
Pathological stage	IA	24(46.2)	19(79.2)	5(20.8)	1.000
	non-IA	28(53.8)	23(82.1)	5(17.9)	
	I-II	41(78.8)	33(80.5)	8(19.5)	1.000
	III-IV	11(21.1)	9(81.8)	2(18.9)	
Tumor size (mm)	mean (SD)	24.9(12.9)	25.4(12.3)	22.6(15.6)	0.542
β_2AR levels	high	10(19.2)	5(50.0)	5(50.0)	0.021

[1]Hospital anxiety and depression scale; [2]p-values based on Chi-square test or Fisher's exact test when expected cell counts are less than 5.

Supplementary Table 2. Association[1] between psychological distress and tumor cell β2AR levels (without adjustments).

		% patients with high tumor cell β2AR levels	OR[1]	95%CI[2]	p-value[3]
**Distress**					
	HADS[4]-T score				
	<13	11.9	ref		
	$\geq$13	50.0	7.4	1.6-37.4	0.011
	HADS[4]-D score				
	<5	7.1	ref		
	$\geq$5	33.3	6.5	1.4-46.8	0.028
	HADS[4]-A score				
	<7	15.0	ref		
	$\geq$7	33.3	2.8	0.6-12.5	0.168

[1]Multiple regression analysis; [2]Confidence interval; [3]P-values based on Chi-squared test when expected cell counts are less than 5; [4]Hospital anxiety and depression scale.

Supplementary Table 3. Association[1] between psychological distress and tumor cell β2AR levels: a sensitivity analysis that excluded six participants with psychological distress (HADS) data available after lung cancer diagnosis but not before.

	% patients with high tumor cell β2AR levels[2]	OR[1]	95%CI[3]	p-value[4]
**Psychological distress**				
HADS[5]-T score				
<13	9.5	ref		
≥13	43.9	7.5	0.9-78.0	0.066
HADS[5]-D score				
<5	3.8	ref		
≥5	28.8	10.2	1.5-207.0	0.042
HADS[5]-A score				
<7	10.8	ref		
≥7	29.7	3.5	0.5-29.5	0.225

[1]Multiple regression analysis, adjusted for age and sex; [2]Adjusted for age and sex; [3]Confidence interval; [4]P-values based on Chi-squared test when expected cell counts are less than 5; [5]Hospital anxiety and depression scale.

Supplementary Table 4. Association[1] between psychological distress with tumor cell β_2AR levels: a sensitivity analysis that excluded four participants who received neo-adjuvant treatment before surgery.

	% patients with high tumor cell β_2AR levels[2]	OR[1]	95%CI[3]	p-value[4]
**Psychological distress**				
HADS[5]-T score				
<13	10.7	ref		
$\geq$13	54.8	10.1	1.5-91.4	0.022
HADS[5]-D score				
<5	7.6	ref		
$\geq$5	31.7	5.7	1.2-41.8	0.047
HADS[5]-A score				
<7	14.1	ref		
$\geq$7	35.2	3.3	0.5-25.0	0.225

[1]Multiple regression analysis, adjusted for age and sex; [2]Adjusted for age and sex; [3]Confidence interval; [4]P-values based on Chi-squared test when expected cell counts are less than 5; [5]Hospital anxiety and depression scale.

Supplementary Table 5. Association[1] between psychological distress with tumor cell β_2AR levels: a sensitivity analysis that excluded 11 participants with pathological stage III-IV.

	% patients with high tumor cell β_2AR levels[2]	OR[1]	95%CI[3]	p-value[4]
**Psychological distress**				
HADS[5]-T score				
<13	11.2	ref		
$\geq$13	55.8	10.0	1.5-94.5	0.025
HADS[5]-D score				
<5	9.1	ref		
$\geq$5	31.6	4.6	0.90-35.0	0.086
HADS[5]-A score				
<7	14.9	ref		
$\geq$7	36.8	3.3	0.5-25.6	0.228

[1]Multiple regression analysis, adjusted for age and sex; [2]Adjusted for age and sex; [3]Confidence interval; [4]P-values based on Chi-squared test when expected cell counts are less than 5; [5]Hospital anxiety and depression scale.
